# IL-17 and TNF-β: Predictive biomarkers for transition to psychosis in ultra-high risk individuals

**DOI:** 10.3389/fpsyt.2022.1072380

**Published:** 2022-12-16

**Authors:** Lijun Ouyang, David Li, Zongchang Li, Xiaoqian Ma, Liu Yuan, Lejia Fan, Zihao Yang, Zhenmei Zhang, Chunwang Li, Ying He, Xiaogang Chen

**Affiliations:** ^1^Department of Psychiatry, National Clinical Research Center for Mental Disorders, The Second Xiangya Hospital of Central South University, Changsha, China; ^2^Hunan Key Laboratory of Psychiatry and Mental Health, Mental Health Institute of Central South University, China National Technology Institute on Mental Disorders, Hunan Technology Institute of Psychiatry, Changsha, Hunan, China; ^3^Department of Radiology, Hunan Children’s Hospital, Changsha, China

**Keywords:** ultra-high risk individual, schizophrenia, inflammation, cytokines, Drug-naïve first-episode

## Abstract

**Background:**

Dysregulation of immunity, such as levels of inflammatory factors, has been regarded as a sign of schizophrenia. Changes in cytokine levels are not only described in the early onset of disease, but also observed in ultra-high risk (UHR) individuals. This study aimed to investigate the potential of cytokines as biomarkers for psychotic disorders and in individuals at UHR of developing a psychotic disorder in the future.

**Methods:**

The Luminex liquid chip technology was used to detect the concentrations of Interferon-gamma (INF-γ), Interleukin (IL)-2, Interleukin (IL)-4, Interleukin (IL)-6, Interleukin (IL)-17, Interleukin-1beta (IL-1β), and Tumor Necrosis Factor-beta (TNF-β) in the plasma of all subjects. Meanwhile, the plasma level of Tumor Necrosis Factor-Alpha (TNF-α) was measured with the enzyme-linked immunosorbent assay (ELISA) kits. Then, the levels of these cytokines were compared among patients with Drug-naïve first-episode schizophrenia (FES; *n* = 40), UHR population (UHR; *n* = 49), and healthy controls (HCs; *n* = 30). Baseline cytokine levels were compared among UHR individuals who later transitioned (UHR-T; *n* = 14), those who did not transition (UHR-NT; *n* = 35), and HCs (*n* = 30).

**Results:**

Our analysis results showed that IL-1β levels were significantly higher in UHR group than HC group (*p* = 0.015). Meanwhile, TNF-α concentration was significantly increased in FES group compared with HC group (*p* = 0.027). IL-17 (*p* = 0.04) and TNF-β (*p* = 0.008) levels were significantly higher in UHR-T group compared with UHR-NT group.

**Conclusion:**

In conclusion, our findings suggest that the immuno-inflammatory activation level is increased in the early stage of psychosis before psychotic conversion and the Drug-naïve FES. IL-1β and TNF-α are the representatives of the specific biomarkers for UHR and FES, respectively. IL-17 and TNF-β may be the potential selective predictive biomarkers for future transition in UHR individuals.

## 1 Introduction

Schizophrenia is a severe psychiatric disorder that severely impairs an individual’s social function, and it can induce disability and high expenses for patients for many years (1, 2). Schizophrenia is characterized by the intense clusters of symptoms, positive and negative symptoms, cognitive deficits, and both affective and aggressive clusters of symptoms (3). Early diagnosis and intervention of schizophrenia is the key to improving the clinical and functional outcomes of the disease (4). Over the last few decades, increasing attention has been paid to people presenting with potentially prodromal symptoms of psychosis (5, 6). It is reported in some research that, approximately 30% of the ultra-high risk (UHR) population will transition to psychosis within 2 years of follow-up (7–9). Therefore, identifying the biomarkers with convenient detection methods and clinical applications has become the focus and challenge of the current research.

The etiology of schizophrenia, though it remains unclear, is hypothesized to be related to the dysfunction of neurotransmitters and neurodevelopment (10, 11). The recent new theories of biological contributors to psychosis tend to concentrate on the role of immune dysfunction (12–14). Molecular genetic studies show that the histocompatibility complex (MHC) is closely associated with schizophrenia. The MHC gene, which is located on chromosome 6, is tightly linked to immunity (15). Sekar et al. demonstrated the impact of a particular mutation of the Complement 4 (C4) gene, which produced the complement protein C4, on synaptic pruning during the crucial stages of brain development, thus increasing the risk of schizophrenia (16). Evidence has identified aberrant immune function not only in the earliest stage of illness, but also before the onset of the disease (17). Some studies indicate that adjuvant treatment with immunomodulatory agents may be beneficial for improving clinical symptoms and social function (18–20). These results provide solid evidence that immunological dysfunction is a key player in the pathophysiology and etiology of psychiatric disorders.

Various cytokines have important functions in both adaptive and innate immune responses. Specifically, the key cytokines related to innate immunity include interleukin-1 beta (IL-1β), interleukin-6 (IL-6), tumor necrosis factor-Alpha (TNF-α), interferon- gamma (IFN-γ), interleukin-2 (IL-2), interleukin-8 (IL-8), interleukin-12 (IL-12), and tumor necrosis factor-beta (TNF-β) from T helper-1 (Th-1) cells, whereas the major cytokines involved in adaptive immunity are interleukin-4 (IL-4), interleukin-5 (IL-5), and interleukin-10 (IL-10) from T helper-2 (Th-2) cells (21, 22). Among them, IFN-γ, TNF-β, and IL-6 may be vital for the activation of Th-1 immunity and the subsequent inflammatory response. The main component of the Compensatory Immune-Regulatory Reflex System (CIRS), IL-4 (the cytokine produced by Th-2 cells), may have immune-regulatory effects (23). Abnormal cytokine levels have already been identified in first-episode psychosis (FEP) (24–26). Moreover, the interleukin-17 (IL-17) axis plays crucial roles in the pathogenesis of several mental disorders (27). As reported in a recent meta-analysis, the levels of IL-6, TNF-α, IFN-γ, IL-17, and transforming growth factor-beta (TGF-β) elevate in FEP compared with healthy controls (HCs) (28). In addition, several studies have demonstrated the systemic alterations of cytokine levels in UHR group, while the results remain controversial (29). A meta-analysis of studies on inflammatory biomarkers reports that IL-6 level increases in UHR group compared with control group, while interleukin-1β (IL-1β) level decreases in UHR group compared with control group (30). As not all UHR individuals will develop psychosis, it is imperative to identify inflammatory biomarkers that are significantly up-regulated in UHR individuals who later develop psychosis (UHR-T) (31). In a recent meta-analysis, the baseline levels of IL-1β, IL-7, IL-8, matrix metalloproteinase (MMP)-8, cortisol, albumin, and salivary cortisol are measured as predictors for UHR-T vs. UHR individuals who later did not transition (UHR-NT) (32). However, another research exhibits an unimportant trend for the higher blood IL-12, IL-1β, and IL-6 levels in converters vs. non-converters (33). The results are inconsistent due to cytokine alterations and are widely affected by potential confounders, including antipsychotics, smoking, and assay methodology.

Some relevant studies have been reported, but they are associated with some limitations, like the small number of cytokine types involved and the focus on a particular stage of schizophrenia. Moreover, many study subjects have received medications, while drug interference with inflammatory factors cannot be excluded. Based on the previously published studies, the present study tested the blood cytokine levels in UHR, Drug-naïve first-episode schizophrenia (FES), and HC groups. Thereafter, these cytokine concentrations were compared between UHR-T and UHR-NT subjects through a 2-year follow-up, aiming to identify cytokines as the potential biomarkers for high-risk psychosis and transition to psychotic disorder. It was hypothesized that pro-inflammatory analytes were up-regulated in early psychosis subjects than in controls and in UHR-T group than in UHR-NT group. Moreover, this study also attempted to determine whether specific changes of cytokine levels in UHR might predict the transition to psychosis.

## 2 Materials and methods

### 2.1 Participants

In this study, FES and UHR subjects were recruited from the outpatient clinics and inpatient units of the Second Xiangya Hospital of Central South University, whereas HC subjects were from the local community in the same region through advertisements. FES subjects developed acute psychosis. All participants were between 13 and 30 years old. Every participant received structured interviews conducted by experienced psychiatrists. This study was approved by the Ethics Committee of the Second Xiangya Hospital of Central South University and performed following the Declaration of Helsinki. All participants were aware of the risks and benefits of this study. Written informed consent was obtained from each subject.

The inclusion criteria for FES were the same as the diagnostic standard of schizophrenia from the fifth edition of the Diagnostic and Statistical Manual of Mental Disorders (DSM-V) (34). Using the Positive and Negative Symptom Scale (PANSS), the symptoms of FES patients were assessed (35). The Structured Interview was adopted for screening all UHR subjects for Prodromal Syndromes (SIPS). As a semi-structured interviewing method, SIPS is created by the PRIME Research Group of Yale University in the US to identify mental risk syndromes. Due to its high reliability and validity, it has been most extensively utilized both at home and abroad. Four elements make up its scale of prodromal symptoms (SOPS). To be specific, on a scale of 0 (no anomaly) to 6 (severe psychotic symptoms), the risk symptoms of psychosis and other symptoms from the last few months are classified as positive symptoms (P), negative symptoms (N), disintegrating symptoms (D), and general symptoms (G) (36). The UHR subjects satisfied the criteria for one of the three subsets, namely, Brief Intermittent Psychotic Syndrome (BIPS), Attenuated Positive Symptom Syndrome (APSS), and Genetic Risk and Deterioration Syndrome (GRDS). All HCs were confirmed to have no family history of psychiatric disorder in their first-degree relatives. The subject exclusion criteria included the history of being diagnosed with any psychiatric disorders and receiving corresponding treatments (including antipsychotics, antidepressants, and mood stabilizers), history of disturbance of consciousness for more than 5 min, major head trauma, history of serious organic diseases (like stroke and heart failure), history of immune system diseases (including bacterial or virus infection, systemic lupus erythematosus), and usage of anti-inflammatory drugs, immunosuppressive drugs or any other medications (oral or injectable) during the last 3 months.

UHR subjects were followed up at regular intervals to identify those who later transitioned to psychosis (UHR-T) and those who did not (UHR-NT). The transition to psychosis was defined by any DSM-V for a mental illness. Telephone assessments were performed monthly during the 2-year follow-up period, including the items of severity, frequency, duration of symptoms and mood, and sleep. The presence of a psychotic syndrome (POPS) questionnaire can exclude previous or current psychotic syndromes, and the diagnosis needs to be established in both (A) and (B). (A) Positive symptoms reach a certain level (score: level 6), such as abnormal thinking content (suspicion, delusion of victimization, delusion of exaggeration), perceptual abnormalities with a low degree of hallucination, and incoherent or incomprehensible speech; (B) at least one of the symptoms meeting criteria (A) occurs 4 days a week on average and lasts 1 h, with a duration of more than 1 month, or the symptoms are seriously confusing and dangerous. Transition to psychosis was comprehensively assessed every 3 months in the first year, every 6 months in the second year, and at the moment of transition.

Both assessments were conducted by two psychiatrists who passed conformance training with an internal conformance coefficient of 0.90 each.

### 2.2 Blood collection and analyses

All blood samples were collected at the baseline visit. Every participant was instructed to avoid caffeine and alcohol consumption, and physical exercise for at least 30 min before any blood sampling. After overnight fasting, blood was sampled from 8:00 a.m. to 10:00 a.m. The blood sample was later centrifuged at 3,000 g for 10 min, and the plasma was stored in a deep freezer (–80°C) before use. The Luminex assay was conducted to measure IL-1β, IL-2, IL-4, IL-6, IL-17, INF-γ, IL-10, IL-8, IL-12, and TNF-β levels at the same time following the manufacturer’s instructions. Both patient and reference serum samples were incorporated in each multi-bioassay plate. In addition, TNF-α level was measured by the high-sensitivity enzyme-linked immunosorbent assay (ELISA) kits in line with the manufacturer’s protocols. Standard curves were prepared by using 4- or 5-PL logistic regression, and data were investigated by the Luminex Flexmap 3D system. A total of 10 proteins were measured with the intra- and inter-assay coefficient of variance of < 10%. Meanwhile, three proteins (IL-10, IL-12, and IL-8) were excluded due to > 80% values below the limit of detection (LOD). For the specific assay, values below the LOD were replaced with the LOD. Six participants (including five FES and one UHR) were excluded because their samples failed quality control.

### 2.3 Statistical analysis

All statistical analyses were conducted with SPSS version 20, and an alpha level of 0.05 was adopted for all analyses. Group differences in age, sex, education, SIPS scores, and PANSS scores were assessed by independent-sample *t*-tests, chi-square test, and one-way analysis of variance (ANOVA). The mean concentration of each biomarker was log-transformed to decrease the data variability and make data more closely conform to the normal distribution. One-way ANOVA was conducted to assess group differences in biomarker concentrations, while *post hoc* comparisons were conducted by using the Bonferroni procedure. False Discovery Rate (FDR) correction was performed for each *p*-value to lower the false positive rate caused by multiple comparisons. The associations of baseline cytokines levels (IL-17, TNF-β), SIPS-N score, and outcomes of UHR subjects (UHR-T and UHR-NT) in 2 years were explored with binary logistic regression.

## 3 Results

### 3.1 Demographic and clinical characteristics

Initially, 142 participants were recruited in the project (including 45 FES subjects, 67 UHR subjects and 30 HCs). However, 23 participants were excluded because their samples failed the quality control criteria (*n* = 6, 5 from FES group and 1 from UHR group), dropped out in the year of follow-up (*n* = 4, all from UHR group), and insufficient follow-up time (*n* = 13, all from UHR group). Finally, 119 participants were included in the analysis, including 40 patients with Drug-naïve FES, 14 with UHR-T, 35 with UHR-NT, and 30 HCs. Table 1 displays the detailed characteristics of all participants. Finally, the clinical outcomes of 49 UHR subjects were obtained. There was no significant difference (*p* > 0.05) in age, gender, or education among the FES, UHR, and HC groups. The detailed demographic and clinical characteristics between UHR-T and UHR-NT groups were compared, as shown in Supplementary Table 1.

**TABLE 1:** Demographic and clinical characteristics.

	FES (*n* = 40)	UHR (*n* = 49)	HC (*n* = 30)	F/x2	*p*
Age (years, mean ± SD)	19.85 ± 5.05	18.33 ± 3.48	19.00 ± 2.92	1.624	0.202
Sex (male/female)	23/17	24/25	21/9	3.360	0.186
Education (years, mean ± SD)	11.08 ± 2.72	10.96 ± 2.56	12.17 ± 2.34	2.276	0.107
SIPS-P score		11.76 ± 5.68			
SIPS-N score		11.72 ± 6.46			
SIPS-D score		3.98 ± 2.42			
SIPS-G score		4.86 ± 2.96			
PANSS-P score	21.31 ± 7.81				
PANSS-N score	25.24 ± 8.59				
PANSS-G score	40.38 ± 8.50				
PANSS-T score	87.02 ± 18.32				

The significance level is 0.05; only significant differences between groups are presented for *post hoc* comparisons. HC, healthy controls; UHR, ultra-high-risk of psychosis; FES, Drug-naïve first-episode schizophrenia; PANSS, Positive and Negative Symptoms Scale; PANSS-P, PANSS positive score; PANSS-N, PANSS negative score; PANSS-G, PANSS general psychopathology score; PANSS-T, PANSS total score; SIPS, Structured Interview for Prodromal Syndromes; SIPS-P score, SIPS positive score; SIPS-N score, SIPS negative score; SIPS-D score, SIPS disorganized score; SIPS-G score, SIPS general score.

### 3.2 Cytokine levels among three groups (FES, UHR, HCs)

**FIGURE 1:**
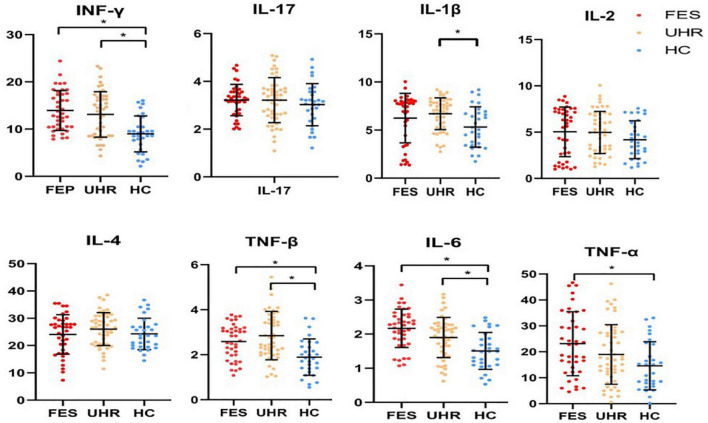
Comparison of cytokines level among the three groups [FES, UHR, HCs, Cytokine concentration (pg/ml)]. Picture shows several significantly different inflammatory analytes between three groups, including IFN-γ, IL-1β, TNF-β, IL-6, and TNF-α, In *Post hoc* comparisons, the Bonferroni procedure revealed that FES patients and UHR subjects exhibited significantly higher mean concentrations of IFN-γ, TNF-β, and IL-6 than HC subjects. Besides, IL-1β concentration was significantly higher in UHR group than HC group, and TNF-α concentration remarkably increased in FES group relative to HC group. *The difference is significant with a *p*-value of less than 0.05. HC, healthy controls; UHR, ultra-high risk of psychosis; FES, Drug-naïve first-episode schizophrenia.

In our study, differences in the concentrations of several inflammatory analytes were statistically significant among the three groups (Figure 1 and Table 2), including IFN-γ, IL-1β, TNF-β, IL-6, and TNF-α. In *Post hoc* comparisons, the Bonferroni procedure revealed that FES patients and UHR subjects exhibited significantly higher mean concentrations of IFN-γ, TNF-β, and IL-6 than HC subjects. IL-1β concentration was significantly higher in UHR group than HC group, and TNF-α concentration was significantly increased in FES group compared with HC group.

**TABLE 2:** Cytokine levels among three groups (FES, UHR, and HCs).

Cytokine (pg/ml, mean ± SD)	FES	UHR	HC	*F*	*p*	FDR
INF-γ	13.95 ± 4.22	13.11 ± 4.93	8.99 ± 3.79	11.750	0.000	0.001*
IL-17	3.22 ± 0.65	3.21 ± 0.94	3.03 ± 0.88	0.570	0.567	0.567
IL-1β	6.25 ± 2.57	6.69 ± 1.65	5.32 ± 2.10	4.133	0.018	0.036
IL-2	5.04 ± 2.68	4.97 ± 2.27	4.19 ± 2.05	1.349	0.264	0.352
IL-4	24.07 ± 7.22	26.02 ± 6.03	24.26 ± 5.78	1.236	0.294	0.336
TNF-β	2.59 ± 0.77	2.85 ± 1.07	1.89 ± 0.81	10.299	0.000	0.001*
IL-6	2.17 ± 0.56	1.90 ± 0.59	1.51 ± 0.54	11.596	0.000	0.001*
TNF-α	23.17 ± 12.33	18.98 ± 14.10	14.62 ± 13.28	3.634	0.029	0.04*

*The difference is significant with a *p*-value of less than 0.05; only significant differences between groups are presented for *post hoc* comparisons. HC, healthy controls; UHR, ultra-high-risk of psychosis; FES, drug-naïve first-episode schizophrenia; FDR, false discovery rate.

### 3.3 IL-17 and TNF-β levels were higher in the UHR-T group than in the UHR-NT groups

**FIGURE 2:**
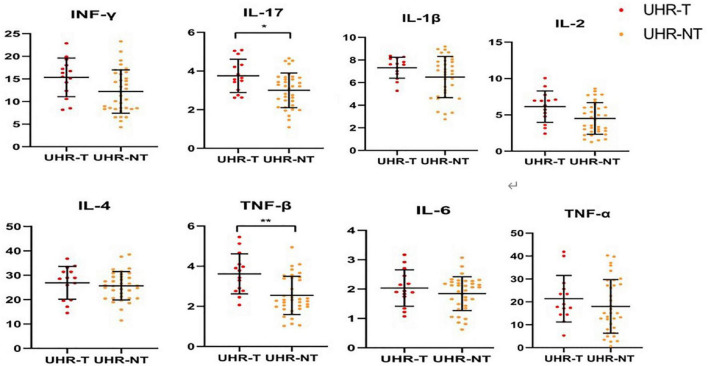
Comparison of the levels of cytokines among the two groups [UHR-T and UHR-NT, Cytokine concentration (pg/ml)]. IL-17 levels were significantly higher in UHR-T group than UHR-NT group [*p* = 0.04, 3.750.86 vs. 3.000.88 pg/ml]. TNF-βlevels were significantly higher in UHR-T group than UHR-NT group [*p* = 0.008, 3.621.00 vs. 2.540.95]. *The difference is significant with a *p*-value of less than 0.05. **The difference is significant with a *p*-value of less than 0.01. UHR-NT, ultra-high-risk without transition; UHR-T, ultra-high-risk with transition.

In our study, the concentrations of two inflammatory analytes, including IL-17 (*p* = 0.04) and IL-1β (*p* = 0.008), were of statistical differences among different groups after FDR correction (Figure 2 and Table 3). The concentrations of IL-17 and TNF-β were significantly up-regulated in UHR-T compared with UHR-NT groups.

**TABLE 3:** IL-17 and TNF-β levels were higher in the UHR-T group than in the UHR-NT group.

Cytokine (pg/ml, mean ± SD)	UHR-T	UHR-NT	*t*	*p*	FDR
INF-γ	15.35 ± 4.27	12.21 ± 4.78	2.131	0.038	0.076
IL-17	3.75 ± 0.86	3.00 ± 0.88	2.680	0.010	0.040*
IL-1β	7.32 ± 0.92	6.50 ± 1.82	1.594	0.118	0.188
IL-2	6.14 ± 2.16	4.51 ± 2.18	2.382	0.021	0.056
IL-4	26.91 ± 6.74	25.67 ± 5.80	0.648	0.520	0.520
TNF-β	3.62 ± 1.00	2.54 ± 0.95	3.523	0.001	0.008*
IL-6	2.04 ± 0.62	1.85 ± 0.58	1.023	0.312	0.416
TNF-α	21.39 ± 10.18	18.03 ± 14.39	0.833	0.409	0.467

*The difference is significant with a *p*-value of less than 0.05. UHR-NT, ultra-high-risk without transition; UHR-T, ultra-high-risk with transition; FDR, false discovery rate.

### 3.4 The effects of N-scores, IL-17, and TNF-β levels on the conversion of psychosis

In this study, binary logistic regression was performed to assess the effects of N-scores, IL-17, and TNF-β levels on the conversion of psychosis in UHR subjects. Our results showed that the logistic model was statistically significant (χ2 = 16.517, *p* < 0.001). The model correctly classified 79.6% of the study subjects. The model sensitivity and specificity were 42.9 and 94.3%, whereas the positive predictive value (PPV) and negative predictive value (NPV) were 75 and 80.5%, respectively. Furthermore, the difference in TNF-β concentration was statistically significant among the three independent variables in the model. The risk of transition increased by 1.505 folds per unit increase in TNF-β (Table 4).

**TABLE 4:** The effects of N-scores, IL-17, and TNF-β levels on the conversion of psychosis.

		B	S.E.	Wald	Sig	Exp (B)	95% C. I. for EXP (B)	
							Lower	Upper
Step 1^a^	SIPS-N score	0.084	0.064	1.705	0.192	1.087	0.959	1.233
	IL-17	0.826	0.468	3.107	0.078	2.283	0.912	5.719
	TNF-β	0.989	0.434	5.181	0.023*	2.688	1.147	6.296
	Constant	–7.811	2.428	10.349	0.001	0.000		

*The difference is significant with a *p*-value of less than 0.05; SIPS-N score, SIPS negative score; IL-17, Interleukin-17; TNF-β, Tumor necrosis factor; ^a^Variable(s) entered on step 1: SIPS-N score, IL-17, TNF-β.

## 4 Discussion

This study investigated cytokines as the potential biomarkers for FES and UHR, and identified an inflammatory marker for distinguishing UHR-T from UHR-NT subjects during the 2-year follow-up period. A significant finding from our study indicated that the baseline levels of IL-17 and TNF-β were significantly different between individuals who converted and those who did not convert to psychosis. Although some previous studies have explored these biomarkers in patients with UHR or psychosis, our subjects did not take any drugs, making our findings more convincing. Previous studies indicate that drugs can alter the levels of inflammatory factors (37). Additionally, since IL-17 and TNF-β concentrations can be directly measured in most clinical laboratories, this study has important clinical implications since it may contribute to identifying UHR subjects who are at a greater risk of developing psychosis.

In our study, FES group showed significantly increased serum levels of TNF-a, TNF-β, IFN-γ, and IL-6 compared with HC group. UHR group presented higher IL-6, TNF-β, IFN-γ, and IL-1β levels than HC group, supporting the presumption of immune alterations in the early stage and even in the prodromal stage of the disease. Our results showed that, the increased levels of TNF-β and cytokines from the T helper type 1 immune response, particularly IFN-γ and IL-6, might indicate the aggravated pro-inflammatory process, which appeared to be related to the onset of a full-fledged psychotic episode that was apparent not only in our FEP group but also in UHR group. There was no significant difference in inflammatory factors between FES and UHR groups, while TNF-α concentration continuously increased in FES group. TNF-α is a major pro-inflammatory factor that can induce the production of a variety of cytokines (38). These results indicate that inflammatory factor concentrations have already increased in the UHR stage, and inflammation may be continuously aggravated as the disease progresses. Our findings support the microglial theory (39), which postulates that the activation of the central nervous system (CNS) microglia will produce pro-inflammatory cytokines including TNF-a and IFN-γ, resulting in aberrant neurogenesis and neuronal degeneration (40). Therefore, it is speculated that a large number of pro-inflammatory factors are released in UHR population, and a different type of immune response is activated. In this study, the Th1/Th2 balance is found to shift toward Th1 in psychotic patients. Inflammatory factors can induce the increased permeability of the blood-brain barrier (BBB) and activate the central immune system.

Moreover, our results also support the hypothesis of a TH1/TH2 imbalance; for instance, a 2015 systematic review shows the presence of Th2 drift in the serum of schizophrenic patients, while an overall immune imbalance in peripheral blood is dominated by Th1-type cytokines (41). In contrast, Th2-type cytokines have been reported to play a dominant role in schizophrenic patients (42–44). These results are inconsistent, but they have provided strong evidence for the presence of immune disorders in the early stage of schizophrenia. In this study, IL-4 concentrations in UHR and FES groups were not significantly changed compared with HC group, while their concentrations in FES and UHR groups showed a rising trend in relative to HC group. Combined with previous studies, UHR is activated by immune inflammation, leading to the increased production of acute phase proteins, such as MI macrophages (IL-1β, IL-6, TNF-β), which shifts toward Th1 in psychotic patients and then leads to activation of the compensatory immunomodulatory reflex system, resulting in the increased production of IL-4 and other anti-inflammatory cytokines. A new homeostasis set point can be formed between anti-inflammatory and pro-inflammatory cytokines, thereby influencing the development of the disease. Moreover, IL-1β level increased only in UHR group but not in FES group, while TNF-α level elevated only in FES group but not in UHR group. Therefore, it was hypothesized that the observed increases in IL-1β and TNF-α levels might represent the specific biomarkers for UHR and FES, respectively.

IL-17, which is produced by Th17 cells, plays a crucial role in various immune and inflammatory processes (45). Th17 cells entering the central center can directly act on endothelial cells by releasing inflammatory mediators like IL-17 and affect brain development together with microglial cells and other cytokines, finally causing changes in brain structure and function (46–48). Previous findings concerning IL-17 in schizophrenia have been inconsistent. In recent studies, individuals with anti-psychotic naïve psychosis show elevated IL-17 levels in the peripheral blood (49). Moreover, the reduced variability of IL-17 concentrations has been reported in subjects with psychosis (50, 51). Our results were in line with the recent meta-analysis, revealing no specific changes in IL-17 levels among FEP patients (52). We found that there was no significant difference in IL-17 levels between FES, UHR, and HC groups, but IL-17 levels were high in UHR-T group compared with the UHR-NT group. An explanation for this result is that IL-17 concentration might be influenced by other cytokines. Previous studies show that when IL-6 concentration reaches a certain level, it can co-form an internal environment with TGF-β to promote Th17 cell differentiation and IL-17 release (53). It is well known that RORt mRNA expression is sparked by TGF-a and IL-6. The precursor cells develop into Th17 subtype when RORt is over-expressed. The activation of STAT3, a crucial transcription factor that controls the effective up-regulation of RORt and Th17-associated genes like IL-17, is triggered by IL-6 and IL-21 (54, 55). In addition, IL-17 induces the production of IL-1β, IL-6, and TNF-α by interacting with its receptor, which can thus facilitate the expansion of the inflammatory process (56–59). Recent studies have reported that IL-17 is positively correlated with IL-6 concentration (60). Some studies also discover that IL-4 can down-regulate the expression of IL-17, possibly because that IL-4 induces the up-regulation of TGF-β and the down-regulation of IL-17 expression by activating regulatory T cells (61). The mechanism of action of IL-17 in the psychosis transition remains unknown. Emerging clinical and experimental evidence has suggested that maternal immune activation during pregnancy can lead to long-lasting changes in the developing brain, which last long after the initial inflammatory stressors are terminated; besides, these changes are associated with an increased risk of various psychiatric disorders (62). IL-17 can mediate various aspects of prenatal immune activation (63). At 24 h after poly I:C stimulation and 2 weeks after delivery, the cytokine indexes of mother mice and their progeny were detected, respectively. It was found that the proportion of CD4 + IL-17 cytokine was significantly higher than that in control group, indicating that immune-activated pregnant parents promoted Th17 cell differentiation in the progeny. Moreover, it increased the number of Th17 cells and IL-17 and caused an increased risk of schizophrenia in the offspring (64, 65).

Another important finding was that TNF-β levels were significantly different between individuals who converted and those who did not convert to psychosis. Furthermore, there was a statistically significant difference in plasma TNF-β levels among FES, UHR and HC groups. Previous studies have paid little attention to the potential role of elevated TNF-β in inducing the risk of a psychotic disorder. One study shows that there are no specific changes in TNF-β levels among subjects at high risk of psychosis (30) while Karanikas et al., who measured 12 circulating cytokines including TNF-β in males at the prodromal stage of psychosis, revealed that only IL-4 levels were significantly increased in UHR subjects than in HCs and schizophrenia patients (66). TNF-β is produced by lymphocytes, activated T cells and B cells, which has an important effect on cell proliferation, differentiation, lipid metabolism and neurotransmitter transmission. It has similar biological activity and effect on TNF-α (67). When bound to TNFR1, it induces the activation of NF-κB, p38α and MK2, promotes the transcription of target genes, the stability and translation of mRNA, and triggers the inflammatory response and cell death (68). Studies have found that the high level of TNF-β is a key acting factor in autoimmune diseases, and schizophrenia is closely related to autoimmune diseases. For example, a large sample study in Denmark based on 7,704 people found that the lifetime prevalence of autoimmune diseases in SCZ patients was 50%. Conversely, the relative risk of SCZ was increased by 45% if there was a history of autoimmune disease (69). The increase in TNF- levels in the UHR-T group might indicate a defective autoimmune mechanism in these people.

In addition, our results were consistent with previous studies that people with FES and a confirmed diagnosis of schizophrenia had elevated IL-6 levels in their peripheral blood (70–72) and cerebrospinal fluid (CSF) (73). According to a longitudinal follow-up study, subjects with high serum IL-6 levels at the age of 9 years had a twofold increased risk of psychosis at the age of 18 years old compared with HCs (74). Another previous research showed that compared with those who did not transition, at-risk mental state (ARMS) subjects who experienced psychosis had higher median IL-6 levels (75). However, there was no significant difference in IL-6 levels between the transition and non-transition groups in our study, probably due to the small sample size, and IL-6 was affected by several factors (76). Another explanation may be that IL-6 is not specific to schizophrenia; some studies have shown that IL-6 is altered in both bipolar disorder and depressed patients (77), and that IL-6 is correlated with negative and positive symptoms (78). The difference in IL-6 levels between the transition and non-transition groups in this study might be affected by mood or pathological symptoms. Our results might be explained by the fact that different types of immune response caused different levels of immune factors, while immune factors interacted with each other, and different factors were associated with different symptoms. For example, research has shown that excitation may be mediated by IL-17 and Uric acid (UA) interactions, whereas negative and cognitive effects may be associated with IL-6 and UA (79). To better understand the contributions of cytokines to positive feedback in schizophrenia, further research is needed.

## 5 Limitations

The pathogenesis of schizophrenia is complex, and the abnormal immune inflammatory system may be involved in the development of mental disorders including schizophrenia. Inflammatory cytokines can be transmitted in the immune inflammatory system, which can reflect the occurrence and development of inflammation. Nevertheless, due to the complexity of the immune inflammatory system and the multi-validity of cytokines, the existing clinical findings are inconsistent. In view of the limited number of studies, small cumulative sample size, and diversity of high-risk criteria, our findings should be interpreted with caution. Therefore, our results should be replicated in other samples before definitive conclusions are drawn. Moreover, in our study, the measurement methods of cytokines were inconsistent due to the deficiencies in the accuracy and precision of detection technology. In this regard, more accurate and sensitive technical methods and accurate statistical methods should be adopted for discussion. Finally, due to the multiplicity of the analyzed markers and various potential untested moderators such as the degree of certain psychopathological symptoms, coexisting physical health conditions, smoking habits, and dietary patterns in our study, it was not ruled out that the significant results on IL-17 and TNF-β might be affected by different variables. In the future, more investigations are needed to further clarify the clinical phenomena and the exact pathogenesis of cytokines in mental illness, aiming to explore more specific inflammatory biological markers for the precise diagnosis and treatment of individualized mental diseases.

## 6 Conclusion

This study reveals the immuno-inflammatory dysregulation in the early stage of psychosis before psychotic conversion and the first episode of schizophrenia. IL-17 and TNF-β levels significantly increase in UHR-T group compared with UHR-NT group, suggesting that IL-17 and TNF-β may be the potential selective predictive biomarkers for future transition in UHR individuals. In addition, IL-1β and TNF-α are identified as the representatives of the specific biomarkers for UHR and FES, respectively.

## Data availability statement

The raw data supporting the conclusions of this article will be made available by the authors, without undue reservation.

## Ethics statement

The studies involving human participants were reviewed and approved by the Ethics Committee of the Second Xiangya Hospital of Central South University. Written informed consent to participate in this study was provided by the participants’ legal guardian/next of kin.

## Author contributions

LO, YH, and XC designed the study, analyzed, and discussed the experimental result. LY, LF, CL, ZZ, ZY, XM, and ZL collected the samples and clinical information. LO wrote the first draft of the manuscript. All authors contributed to the article and approved the final manuscript.
