# Supplementary material for: IL-17 and TNF-β: Predictive biomarkers for transition to psychosis in ultra-high risk individuals
Source: Front Psychiatry. 2022 Dec 16;13:1072380. doi: 10.3389/fpsyt.2022.1072380 (PMC9800867; doi:10.3389/fpsyt.2022.1072380)
Supplement: Supplementary file 1 [file Table_1.docx]

Supplementary Material

**Table S. Demographic features of UHR-T、UHR-NT group and HC group**

There were no significant differences (p > 0.05) in age, gender, and education between the 3 groups (UHR-T, UHR-NT, and HC). The UHR-T group showed a significantly increased SIPS-N score compared to the UHR-NT group The detailed clinical characteristics of the participants are shown in (Table S).

|  | UHR-T | UHR-NT | HC | F/x^2^ | *p* |
| --- | --- | --- | --- | --- | --- |
| Age(years,mean±SD) | 18.86±2.98 | 18.11±3.68 | 19.00±2.92 | 1.624 | 0.202 |
| sex(male/female) | 8/6 | 16/19 | 21/9 | 3.877 | 0.143 |
| education(years,mean±SD) | 10.57±2.41 | 11.11±2.64 | 12.17±2.34 | 2.276 | 0.107 |
| SIPS-P score | 10.71±5.76 | 12.31±5.70 |  | -0.886 | 0.38 |
| SIPS-N score | 14.86±6.95 | 10.54±5.99 |  | 2.716 | 0.035 |
| SIPS-G score | 4.57±2.84 | 3.80±2.23 |  | 1.005 | 0.32 |
| SIPS-P score | 4.71±2.40 | 4.94±3.23 |  | -0.239 | 0.812 |
